# Bridging the divide: a model-data approach to Polar and Alpine microbiology

**DOI:** 10.1093/femsec/fiw015

**Published:** 2016-01-31

**Authors:** James A. Bradley, Alexandre M. Anesio, Sandra Arndt

**Affiliations:** 1 Bristol Glaciology Centre, School of Geographical Sciences, University of Bristol, BS8 1SS, UK; 2 BRIDGE, School of Geographical Sciences, University of Bristol, BS8 1SS, UK

**Keywords:** models, model-data integration, Polar and Alpine microbiology, interdisciplinary approach, quantitative methods

## Abstract

Advances in microbial ecology in the cryosphere continue to be driven by empirical approaches including field sampling and laboratory-based analyses. Although mathematical models are commonly used to investigate the physical dynamics of Polar and Alpine regions, they are rarely applied in microbial studies. Yet integrating modelling approaches with ongoing observational and laboratory-based work is ideally suited to Polar and Alpine microbial ecosystems given their harsh environmental and biogeochemical characteristics, simple trophic structures, distinct seasonality, often difficult accessibility, geographical expansiveness and susceptibility to accelerated climate changes. In this opinion paper, we explain how mathematical modelling ideally complements field and laboratory-based analyses. We thus argue that mathematical modelling is a powerful tool for the investigation of these extreme environments and that fully integrated, interdisciplinary model-data approaches could help the Polar and Alpine microbiology community address some of the great research challenges of the 21st century (e.g. assessing global significance and response to climate change). However, a better integration of field and laboratory work with model design and calibration/validation, as well as a stronger focus on quantitative information is required to advance models that can be used to make predictions and upscale processes and fluxes beyond what can be captured by observations alone.

## Abstract (precis)

Mathematical modelling is an underexploited resource in Polar and Alpine microbiology, and can be integrated with empirical studies to better understand, quantify and predict microbial dynamics.

## INTRODUCTION

The cryosphere comprises a complex network of interacting biological, physical and geochemical processes. Unsurprisingly, the means by which these processes are studied differ greatly in terms of the techniques, the tools and methodologies used, and the scale at which they are resolved. In the physical sciences, modelling has traditionally been an integral part of the scientific method, and recent advances in the understanding of physical processes that characterize Polar and Alpine regions have emerged from such integrated model-data approaches. Examples include the changing mass balance of glaciers and ice sheets over a timescale of days to millennia (Ritz, Rommelaere and Dumas 2001; Hanna *et al.*^2013^), the gravimetric flow of glaciers (Bueler and Brown 2009; Larour *et al.*^2012^) and the contribution of ice melt to past and future sea level (Price *et al.*^2011^; Gillet-Chaulet *et al.*^2012^; Nick *et al.*^2013^; Clarke *et al.*^2015^). Similarly, numerically-based approximations of the physics and chemistry of the Polar oceans, sea-ice and wetlands, and the atmosphere have allowed scientists to improve understanding of the processes that dominate these systems across scales and to make future predictions (Bailey and Lynch 2000; Wu, Budd and Allison 2003; Valkonen, Vihma and Doble 2008; Seroussi *et al.*^2014^). These large-scale predictive models are constructed from fundamental physical laws (Blatter 1995; Bueler and Brown 2009; Hindmarsh 2012), and their results can generally be constrained by satellite observations (Moon *et al.*^2012^).

Conversely, the interest in modelling microbial systems in Polar and Alpine regions has been modest. As a result, recent advances in microbial ecology in Polar and Alpine regions have largely been driven by field-based sampling and laboratory-based analyses. Leading-edge discoveries have resulted from mostly empirical approaches, such as using genomic and metagenomic techniques to investigate the biodiversity of glacial ecosystems (reviewed by Anesio and Laybourn-Parry 2012) and Polar soils (Neufeld and Mohn 2005; Pearce *et al.*^2012^), *in situ* analyses of seasonally changing snow-packs (Larose, Dommergue and Vogel 2013a,b) and sea-ice (Bowman *et al.*^2012^), and chemical and biological characterization of seasonally and perennially ice-covered lakes (Dolhi *et al.*^2015^). Furthermore, feedbacks between the biological processes on glaciers and ice sheets and their physical properties, such as biologically induced darkening of glacier surfaces (Stibal, Sabacka and Zarsky 2012; Yallop *et al.*^2012^), have been identified through experimental studies as potentially important drivers of the climate system. In the wake of this mostly empirically-based research, quantitative and numerical approaches are lacking. After all, the major research questions of the 21st century, especially in high-latitude regions, are inherently quantitative and require the development of robust upscaling strategies or the ability to make predictions about future responses. Examples include constraining the carbon budget of soils or glacier surfaces (Hodson *et al.*^2007^; Schmidt *et al.*^2008^; Telling *et al.*^2010^), the emission of methane from thawing permafrost (McCalley *et al.*^2014^; Hultman *et al.*^2015^), the role of nitrogen fixation on glacier surfaces (Telling *et al.*^2011^) and the susceptibility of microbial ecosystems to climate change (Deslippe *et al.*^2012^; Karhu *et al.*^2014^). Similarly, a quantitative appreciation of community interactions together with empirical characterization of the microbial community is needed to determine how a microbial community may be structured according to biological interactions and the physical and chemical environment imposed on it. Consequently, fully integrated and interdisciplinary model-data approaches are essential to formulating robust strategies with which to tackle these challenges and advance our understanding. The current lack of such approaches can be partly attributed to the absence of a fundamental common mathematical framework. Whereas many aspects of physical science can be mathematically described by the laws of physics, biological processes must usually be generalized, simplified and to some degree, abstracted. Describing biological systems in a mathematical framework is further complicated by their inherent stochastic nature. However, mathematical models (see Table 1 for definition of terms) in combination with data can be extremely powerful. Models help not only in disentangling the complex process interplay underlying field observations, quantifying processes and fluxes, understanding the interactions of microbes with each other and their environment, testing sensitivities and making scenario-based predictions, but also in identifying gaps in current understanding, informing efficient and effective laboratory and field studies and shaping the direction of future research.

**Table 1.** Glossary of terms.

Term	Definition
Analytical model.	A model for which a set of mathematical equations can be solved analytically (by exploiting known mathematical rules to express one variable in terms of other variables without using numerical computations) to examine the prediction and behaviour of that model (compare with ‘Numerical model’).
Calibration / Tuning.	The process of adjustment of model parameters to obtain a representation of model dynamics (e.g. time-series) that agrees with pre-agreed criteria (usually observational data).
Chaotic dynamics.	A dynamical system with strong dependency on initial conditions, which can make long-term predictions impossible.
Deterministic.	A model in which there are no random events (the same input will always produce the same output).
Differential equation (ordinary or partial).	A mathematical function that relates a function with its derivatives, usually to represent the rate of change and relationships between state variables.
Ecological model.	The use of mathematics to understand and predict ecosystem behaviour.
Individual-based model.	A model of a system of individuals and their environment, where system behaviour arises from individual traits and characteristics of organisms and the environment, and the interactions between them.
Mathematical model.	An equation or set of equations that mathematically describe a system.
Michaelis–Menten/Monod kinetics.	A specific and commonly used model of enzyme kinetics whereby a maximum reaction rate is modulated by substrate concentrations in a saturating form (see Fig. 2) (sometimes referred to as Monod kinetics when applied to microbial growth).
Numerical model.	In contrast to an ‘Analytical model’, a numerical model is a mathematical model that must be solved numerically (using a computational time-stepper) to evaluate model prediction and behaviour.
Parameter.	A value (or measurable factor) that stands for inherent properties of a system component (and may implicitly account for processes that are not explicitly accounted for in the model) that can be varied in calibration/tuning exercises.
Process-based model.	A model that explicitly incorporates aspects of the biological system in a mathematical formulation (compare with ‘Statistical model’).
Sensitivity.	A measure of the dependence of model outputs on values specified in the model formulation (e.g. parameters, initial conditions).
State variable.	A measure of the status of an individual variable in a model (e.g. population biomass and substrate concentration).
Statistical model.	A model that examines distributional properties of data, typically without including any explicit biological processes (compare with ‘Process-based model’).
Stochastic.	A model in which random events play a role (a given input may produce many different outputs).
Uncertainty.	The variability that arises in model output given the uncertainty in the inputs (e.g. parameters).
Validation / verification.	The process of determining that model dynamics accurately represent the developer's conceptual description and specifications, usually by comparison to observational data (that is independent of data used in calibration/tuning).

Here, we briefly introduce the concept of mathematical models and how integrated model-data approaches might be used and applied to some of the most pressing questions in Polar and Alpine microbiological research. We hope that by discussing some of the problems and common criticisms of mathematical models in microbiology, we can enthuse microbiologists working in Polar and Alpine regions to consider, develop and use integrated model-data approaches to explore the microbial dynamics of cold ecosystems. Finally, we stress the importance for future biologically-oriented field and laboratory investigations to carefully consider how measurements are made, such that data may strengthen model design, and validate and inform their predictions in the future.

## MATHEMATICAL MODELS

Mathematical models have played an important role in developing modern ecological theory, and in establishing knowledge about the interactions between the Earth's microbiome and the physical, chemical and biological environment in which they live, in a way that is often not possible using purely empirical approaches (Jessup *et al.*^2004^; Larsen, Hamada and Gilbert 2012). Mathematical modelling is not an end in itself, and there should always be a good reason for using a model. However, there are a number of important constraints to the purely observational approach. It is extremely difficult and often even impossible to disentangle the underlying process interplay for observations that reflect the net process outcome often observed. Furthermore, sampling techniques may disturb the environment to be studied (e.g. distinguishing microbial activity in frozen soils from experimental artefacts). In addition, many environments, including the Polar and Alpine environment, are difficult and expensive to reach and sample, limiting the availability of data on both temporal and spatial scales. Finally, observations are snapshots of a complex, evolving environment and provide sometimes limited information about past dynamics and potential system responses to on-going or projected change. Recognizing these limitations, microbiologists may resort to designing laboratory models (e.g. microcosms) to obtain insights into processes or to make predictions. Mathematical models are very similar to laboratory models, in that, they are simplified representations of the reality that is too complex to easily understand and manage *in situ*. Laboratory experiments are based on conceptual models that, like mathematical models, do not consider all the processes that occur in the environment, but the ones essential to the problem.

Common approaches to modelling microbial ecosystems are listed in Table 2, and are linked to Polar and Alpine applications in the following sections. When deciding which approach to take, one must consider the nature of the research question, the scale that must be resolved (e.g. metabolic, microbe, community and ecosystem), the level of basic knowledge of the system, the available computational power and the demands of the model and the quality of observational data available. Therefore, each unique scientific question likely has several ‘best possible’ solutions integrating models and data. Thus, a major challenge is the exchange of knowledge between modellers and empiricists to design the best strategy for a specific research question. Another major challenge is how to best integrate microbial models with existing biogeochemical and physical models in Polar and Alpine regions. However, we hope that by discussing the fundamental principles of many microbial models and how amenable (or not) they are to different facets of Polar and Alpine microbiology research, and by suggesting means by which these models can be applied to various Polar and Alpine systems, common ground can be found. Ultimately, we hope to convince empiricists to collaborate with modellers and to consider using and developing microbial models themselves, and those communities already modelling physical and/or biogeochemical processes in Polar and Alpine systems to consider incorporating explicit microbial dynamics into their numerical formulations.

**Table 2.** Approaches to modelling microbial dynamics.

Model			Information	Information
approaches	Examples	Formulation	required	provided
Process-based models.	Blagodatsky and Richter (1998), Stapleton *et al.* (2006), Bradley *et al.* (2015)	Differential or partial differential equations. Michaelis-Menten/Monod growth kinetics.	Physiological rates (e.g. specific growth rate, mortality) at prescribed conditions. Initial values.	Numerically solved time-series of state-variables, production and activity rates.
			Forcings (e.g. time-series of environmental conditions).	
Stage-structured population model.	Moorhead *et al.* (2002)	Population life-cycle stages.	Physiological rates (e.g. fecundity, mortality). Forcings (e.g. time-series of environmental conditions).	Population structure and dynamics in relation to environment.
Bioclimatic models.	Steele *et al.* (2011)	Envelope models. Ecological niche models. Species distribution models.	Physiological response to biotic and abiotic factors. Classification of habitat space.	Predicted ecological niche dynamics and species distributions.
Individual-based models.	Ginovart, Lopez and Gras (2005), Hellweger and Bucci (2009), Gras *et al.* (2010)	Spatially and temporally resolved individual organisms.	Predicted metabolism of each cell on a lattice (grid) of environmental parameters and metabolite concentrations.	Predictive power in highly complex and heterogeneous environments.
Energy-based models.	Gonzalez-Cabaleiro, Lema and Rodriguez (2015*)*	System dynamics are regulated by metabolic networks.	Metabolic reaction network. Gibbs free energy of central catabolic reactions.	Product yields of various chemical compounds.
Fitted models.	Schnecker *et al.* (2014)	Structural Equation Models (SEM). Generalized Linear Mixed Models (GLMM).	Comprehensive sampling and data-collection strategy. Extensive meta-data.	Spatial, temporal and geophysical correlations between variables.
		Simultaneous Autoregressive Models (SAR).		

Polar and Alpine regions in particular are ideal environments for the development and application of certain types of local and system-scale models that include an explicit description of microbial dynamics for the following reasons.

### Growth-limiting conditions

By studying life at its limit (e.g. low-temperature and low-nutrient availability), much can be learnt about microbial metabolisms, energy requirements, adaptation and survival strategies. This is relevant, for instance, to our understanding of life refugia during snowball Earth (e.g. Telling *et al.*^2015^) or the potential for life elsewhere in the solar system (e.g. Lamarche-Gagnon *et al.*^2015^; Mikucki *et al.*^2015^). The harsh environmental conditions that are typical of high-latitude and high-altitude regions (cold temperatures, frequent freeze–thaw cycles, low water, low-nutrient availability, high exposure to ultraviolet radiation in the summer and prolonged periods of darkness in winter) limit microbial growth and affect community structure (Cary *et al.*^2010^). Specific mathematical formulations can be easily integrated into process-based models (e.g. Stapleton *et al.*^2006^; Bradley *et al.*^2015^), such as Monod or Michaelis–Menten-type dynamics to describe light or substrate limited growth, and Arrhenius-style formulations (such as Q_10_) to describe temperature dependencies.

### Seasonality—sampling bias

High-latitude regions are characterized by extreme seasonality. Long summers are punctuated by extended periods of 24-h darkness, snow cover and sub-zero temperatures. The majority of biological data is collected during the summer period, compromising its use for annual extrapolations. Process-based, individual-based and energy-based models can be used to explore the dynamics over winter seasons, which are characterized by very different external forcings (e.g. temperature, snow cover and incidence of solar radiation). On the contrary, statistical models, which are primarily driven by data, may not be particularly suited to winter studies due to the sparseness and largely opportunistic nature of empirical observations during Polar night.

### Simple trophic structure

Ecosystems in Polar environments (especially Antarctica) may have a relatively simple trophic structure (compared to many temperate environments) due to their inhospitable environmental conditions inhibiting plant and animal colonization (Bottos *et al*. 2014a). Therefore, Polar microbial communities, such as those inhabiting Antarctic Dry Valley soils, may be more amenable to modelling than, for example, temperate soils, in a process-based model. Process-based models typically represent microbial growth and community interactions by differential or partial differential equations, which reflect and predict behaviour, and differences in physiology between taxonomic or functional groups (represented as separate state variables) are formed mathematically. A system with relatively lower trophic complexity may be categorized into fewer taxonomic/functional groups (and therefore there will likely be fewer state variables and parameters). A model with fewer variables and parameters is often easier to constrain based on empirical data. Individual-based models (whereby individual cells are resolved on a heterogeneous lattice) and stage-structured population models (whereby the life-stage and life-cycle of an organism is explicitly defined) are also well suited to modelling (often highly diverse and complex) microbial communities because there may be fewer niches or interacting variables (which ultimately would need to be constrained by observations).

### Spatial scale

The Arctic and Antarctic biosphere is a geographically expansive area, which is often challenging and expensive to access. This results in relatively patchy data coverage, and therefore, a potentially incomplete picture of system dynamics from spatially and temporally discreet field sampling strategies. Models can bridge scales and interpolate observations. Statistical models can be used to account for differences in microbial communities, making spatial, temporal and geophysical correlations between spatially discreet sampling sites. These types of models can also be used to help design efficient strategies for fieldwork by identifying geographical points of interest or areas of broadly similar dynamics.

### Rapidly changing climate

Ecosystems in Polar regions are likely to be among the most strongly affected by global climate change in the near future (Serreze *et al.*^2000^). Due to the severe biological constraints imposed by the environment, Polar ecosystems are likely to be highly sensitive to climatic changes (e.g. alleviation of temperature-limited growth, disturbance due to changing hydrological regime and invasive species). A process-based modelling approach may be useful to explore the potential responses and vulnerabilities (e.g. tipping points) of Polar ecosystems to global climate change using scenario-based (e.g. IPCC) predictions. Additionally, bioclimatic modelling approaches (whereby the geographic ranges and distributions of organisms are predicted as a function of climate) may be particularly well suited to these problems.

### Genomic potential

Although not unique to Polar and Alpine environments, a wealth of genomic data is starting to become available from high-latitude and high-altitude ecosystems. This offers a new opportunity to construct mathematical models that incorporate microbial function (e.g. genomics and transcriptomics) with biogeophysical processes. The development of mathematical models and the assemblage of molecular datasets have traditionally been distinctly separate in scientific practice; however, recent efforts to integrate models with genomic data (such as gene expression) are promising. For example, Reed *et al.* (2014) developed a new process-based modelling approach whereby oceanic nitrogen dynamics and cryptic sulphur cycling were explored using a model that predicts the rate of functional gene expression alongside biogeochemical and microbial processes (such as chemical concentrations and abundances). This way, the model output and comparison and validation exercises can be integrated with genomic data.

The following paragraphs, alongside Fig. 1, outline the necessary steps involved in a sound modelling approach for Polar and Alpine regions, and highlight opportunities for collaboration and interdisciplinary knowledge exchange between modellers and observationalists.

- Observe the natural system and identify research questions (modeller and observationalist)
  **Figure 1.**
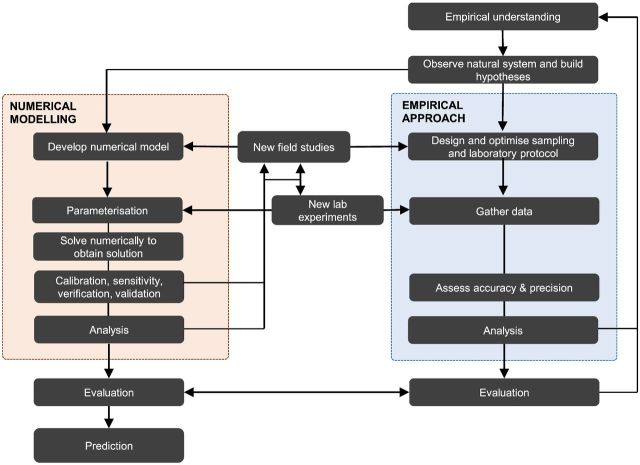
Flowchart illustrating the scientific technique, emphasizing the relationship between a numerical modelling approach and an empirical approach, and the scope for interdisciplinary collaborations by integrating the two.
  A research question or hypothesis is formulated based on existing knowledge and observations using techniques such as genomics and metagenomics (e.g. Pearce *et al*. 2012) and geochemistry (e.g. Hawkings *et al*. 2015).
- Conceptual model (modeller and observationalist)
  The conceptual model captures the essential components of the system and their interactions. The other aspects of the system are omitted in order to reduce unnecessary complexity and ultimately reduce uncertainty that arises from limited data availability or knowledge. Direct and interdisciplinary collaboration between modellers and empiricists is, at this stage, crucial, in deciding which of the physical, chemical and biological components and processes known to occur in Polar and Alpine systems to include explicitly in models, and which to omit.
- Formulate the mathematical model (modeller)
  The conceptual model is formulated in the form of mathematical expressions. For example, in Arctic tundra soils, a process-based model may be formulated from a set of differential equations that describe the transfers and transformations of major elements (such as carbon, nitrogen, phosphorus and sulphur) through different trophic levels. These trophic levels may be organized into food webs if such dynamics are essential to the accurate representation of the system. For example, the Stapleton *et al*. (2006) soil model resolves individual phyla to include top-down controls on microbial populations (including protozoan and nematode grazers) in a soil ecosystem in Svalbard, based on empirical evidence. Conversely, the SHIMMER soil model (Bradley *et al*. 2015), which was designed to predict soil development rather than capture trophic interactions, lumps top-down controls (including predation and viruses) into a single expression for the sake of maintaining a manageable level of model complexity. Thus, biotic feedbacks such as predation, which are shown to be important from empirical studies, may be deliberately omitted from some models or lumped together with other processes in order to simplify system dynamics. In formulating microbial community interactions, mathematical notation may be used to simulate observed phenomena such as substrate-limited growth (usually described by Monod or Michaelis–Menten growth kinetics whereby maximum specific growth rates are modulated by saturation-coefficients (Fig. 2)), temperature dependencies (by Arrhenius-style formulations such as Q_10_), light dependencies and dormancy. Alternatively, in data rich systems (such as some tundra soils), fitted statistical models may be used to determine correlations between components of the system and explain spatial and temporal patterning. For example, structural equation modelling (SEM) is used to predict organic matter decomposition based on microbial community composition in Siberian Arctic soils (Schnecker *et al*. 2014). At this stage, mathematics is just the language; it enables the modeller to develop a framework that helps convey quantitative information and encapsulate relationships mathematically. Empirical observations of microbial processes inform how these processes are formulated.
- Parameterization (modeller and observationalist)
  **Figure 2.**
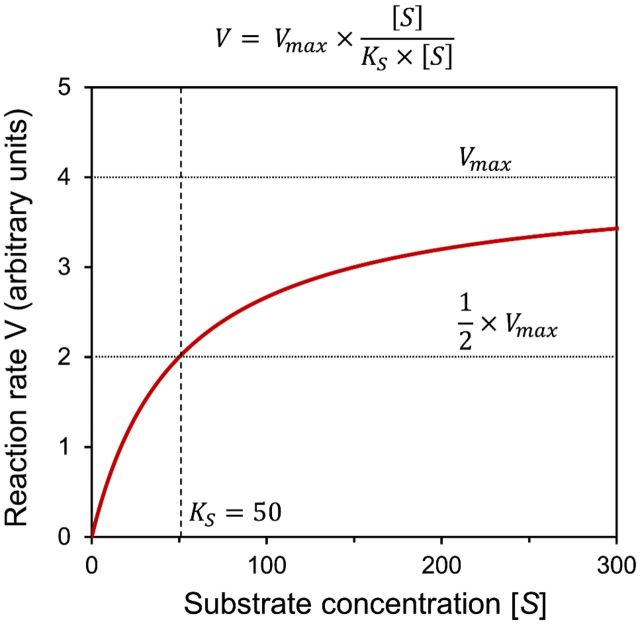
Mathematical formulation and graphical depiction of substrate limited growth with Michaelis–Menten / Monod kinetics. The rate of microbial growth (v) is described by relating the maximum possible growth rate (v_max_) to the concentration of a limiting substrate (S). The constant K_S_ is the substrate concentration at which the growth rate is half of v_max_, and may be derived empirically.
  The mathematical formulation of the model requires the assignment of parameters (see Table 1). Tangible biologically relevant expressions (such as microbial growth rates, efficiency and temperature and light dependency) may be determined empirically using well-designed experimental protocols. Such experiments may be challenging to carry out in the field, especially in Polar and Alpine regions (due to isolation, cleanliness, cold and other practical issues). However, they would provide context *in situ*. Alternatively, laboratory-based mesocosm incubations of samples collected in the field allow conditions to be controlled and specific variables can be isolated. Some parameters are poorly constrained by empirical data. For example, accurate representation of microbial death rate is important since this process strongly influences the size of the necromass pool and thus the availability of organic substrate on which heterotrophic populations depend. However, empirical studies on microbial death and physiological state are lacking (Toal *et al*. 2000). Therefore, modelling these processes (e.g. Blagodatsky and Richter 1998) is challenging. Improved empirical evidence of cell death and dormancy, making use of techniques including direct viable counts, live/dead stains, enzyme/protein synthesis and RNA quantification, will thus enable modellers to improve how these processes are formulated mathematically. More abstract parameter values (that implicitly account for all processes that are not explicitly accounted for in the model) are specific to individual model formulations and have to be determined by fitting the model to observations (see below for calibration). The parameterization of a model thus provides another opportunity for close collaboration.
- Solution (modeller)
  The mathematical model is analytically or numerically solved to provide output (usually as a time-series resulting in complex (or chaotic) transient or steady-state behaviour).
- Calibration, sensitivity, verification and validation (modeller and observationalist)
  Model predictions are constrained by observational data. These include observations of steady-state behaviour, time-dependent values of state-variables, reaction rates or (more recently) gene expression. In order to obtain an acceptable fit between model dynamics and observational data, model adjustment is conducted via calibration. This is usually an iterative process, whereby if the model does not capture the observations, the model may be missing an important process (in which case, the modeller needs to go back to the Development stage, Fig. 1), or require optimization of parameters (in which case, the modeller needs to go back to the Parameterization stage). In addition, the model can also be used to test the sensitivity of model output to variations in mathematical expressions and/or parameters.
- Analysis (modeller and observationalist)
  The model is then applied to predict the behaviour of the system, analyse its dynamics, quantify processes and calculate budgets. Model output must always be interpreted in the context of the model formulation and complexity, so as to not make unjust assumptions about the dynamics of the true natural system. A major advantage of a combined model-data approach in Polar and Alpine microbiology is the ability to separate the various fluxes and rates of biogeochemical processes, which are often only reflected as a net outcome in empirical studies. Not only can the analysis stage further understanding and strengthen evidence, but it may also help to design more appropriate field and observational approaches for studying Polar and Alpine biogeochemical dynamics. For instance, negative results (i.e. the model does not capture the observed features) may indicate an incomplete understanding of the inner-workings of the system, and point towards a missing feature or process, in turn prompting a new direction of empirical field or experimental research.

## MATHEMATICAL MODEL APPLICATIONS IN POLAR AND ALPINE MICROBIOLOGY

Microbiological studies aimed at characterizing Polar and Alpine ecosystems have resulted in a wealth of observational data (Boetius *et al.*^2015^). Despite of the good data availability, many hypotheses tend to be descriptive, rather than quantitative. This is partly due to the complex interactions of biological, geochemical and physical processes that are often obscured by spatial and temporal heterogeneity, stochastic behaviour and transient responses to environmental changes. Despite microbes being known to be major drivers of elemental cycling in the cryosphere (Anesio and Laybourn-Parry 2012), many biogeochemical models do not explicitly account for microbial biomass dynamics, thus assuming that microbial biomass is in a steady state (e.g. Thullner, Van Cappellen and Regnier 2005; Dale *et al.*^2009^; Yang *et al.*^2009^). This is due, first, to the emergence of biogeochemical models from the field of geochemistry and thus their geochemistry-focused approach, second, to the lack of information required to constrain microbial community dynamics and third, the often negligible influence beyond transient timescales. Consequently, there is a unique opportunity to use existing data sets to improve and develop models that can be applied to Polar and Alpine regions with the aim of understanding microbiomes and make accurate predictions about their role in a changing climate scenario.

Previous published studies that have used numerical modelling approaches to explore Polar microbial ecosystems are summarized in Table 3a. These models differ greatly in terms of the spatial and temporal scales that they resolve, and the formulation of microbial and biogeochemical processes. Many of the process-based models listed resolve relatively small spatial scales (cm–m) across a single dimension (time) with no explicit longitudinal or depth component, across relatively short time-scales (e.g. daily to yearly simulation times) (McKane *et al.*^1997^; Moorhead *et al.*^2002^; Stapleton *et al.*^2006^; Bradley *et al.*^2015^). They typically simulate local microbial and nutrient dynamics, and local empirical data are used to inform and validate their predictions. For example, Stapleton *et al.* (2006) described the dynamics of multiple taxa in an Arctic soil mathematically (using Monod-type dynamics), and integrated empirical observations and measurements to understand predation, the Arctic microbial food web and the impacts of anthropogenic nitrogen deposition over relatively short time-scales (days–months). Similarly, Bradley *et al.* (2015) designed a model framework to simulate the development of soil and microbial dynamics in recently exposed glacier forefields over a multidecadal timeframe, using incubations and rate assays to inform parameter values (Frey *et al.*^2010^). These models have proven useful, for example in assessing the relative importance of photosynthetic activity compared to heterotrophic activity in an oligotrophic system, and the role of temperature dependency and dormancy in the stability of microbial populations during winter. Modelling approaches can also be used to explore the sensitivity of a natural system (e.g. biogeochemical cycling and microbial community structure) to natural changes. For example, tipping points can be identified, whereby small perturbations lead to more pronounced changes due to positive feedback mechanisms. This may reveal vulnerable aspects of a system where possible protection or preventative means should be established.

**Table 3.** (a) Present and (b) potential future model applications to Polar and Alpine microbiology.

Ecological		Model	Spatial	Temporal
problem	Reference	type / formulation	scale	scale
(a) Model studies.				
High-Arctic soil microbial, grazing (food-web) and nutrient dynamics.	Stapleton *et al.* (2006), Bradley *et al.* (2015)	Process-based model. Explicit microbial biomass pools. Michaelis–Menten / Monod growth kinetics.	cm^2^–km^2^	Daily–decadal
		Constrained by field and lab observations.		
Arctic tundra carbon and nitrogen dynamics.	McKane *et al.* (1997)	Process-based ecosystem model.	m^2^	Annual
Antarctic lake microbial mat net-ecosystem production.	Moorhead, Schmeling and Hawes (2005)	Bioclimatic model. Environmentally forced ecosystem production.	m	Daily– annual
		No explicit biomass pools.		
Methane accumulation in sub-Antarctic sediments.	Wadham *et al.* (2012)	Depth-resolved numerical hydrate model and reactive continuum model.	Continental	10^3^–10^6^ years
Arctic Soil Organic Matter (SOM) decomposition.	Schnecker *et al.* (2014)	Fitted model (SEM).	Regional	
Global carbon cycle (including high-latitude regions).	Wieder, Bonan and Allison (2013)	Process-based model. Explicit microbial biomass pools. Michaelis–Menten growth kinetics.	Global	Decadal
Dissolved Organic Carbon (DOC) export to Arctic ocean.	Manizza *et al.* (2009)	Ocean general circulation biogeochemical model.	Regional	Monthly
Nematode population structure.	Moorhead *et al.* (2002)	Stage-structured population (life-cycle) model, constrained by lab cultures.	m^2^	Daily
(b) Potential future model applications.				
Chemical budget of a glacier catchment.	Hodson *et al.* (2005)	Bioclimatic model. Process-based model.	Plot (m^2^)—catchment (10^3^–10^6^ m^3^)	Daily–monthly
Snow ecology (e.g. snow algae).	Lutz *et al.* (2014), Lutz *et al.* (2015)	Process-based model (0-D or depth-resolved). Stage-structured population (life-cycle) model.	Plot (m^2^)—catchment (10^3^–10^6^ m^3^)	Daily–monthly
		Gene-centric model.		
Snow biogeochemistry.	Kuhn (2001), Larose, Dommergue and Vogel (2013a,b), Bjorkman *et al.* (2014)	Depth-resolved Reactive Transport Model (RTM).	Plot (m^2^)—catchment (10^3^–10^6^ m^3^)	Daily–monthly
Glacier surface ecology (cryoconite, host-virus interactions).	Fischer *et al.* (2004), Bagshaw *et al.* (2013), Bellas *et al.* (2013)	Predator–prey / Lotka–Volterra model. Process-based model.	Cryoconite hole (cm)—glacier surface (km)	Daily
		Gene-centric model.		
Seasonal changes to high-latitude ecosystem.	Schadt *et al.* (2003), Lipson and Schmidt (2004), Lazzaro, Brankatschk and Zeyer (2012)	Process-based model. Bioclimatic model. Fitted model.	Catchment (10^3^–10^6^ m^3^)	Monthly
Aerobiology over an ice sheet.	Bottos *et al.* (2014b), Pearce *et al.* (2016)	General circulation model coupled to ice surface process-based model.	10^3^km	Daily
Lakes (sub-glacial lakes, ice-covered or open surface lakes, microbial mats).	Christner *et al.* (2014)	Depth-resolved Reactive Transport Model (RTM) coupled to Michaelis–Menten / Monod growth.	m	Daily–decadal
		Gene-centric model.		
Sea-ice ecology and biogeochemistry.	Becquevort *et al.* (2009)	Depth-resolved Reactive Transport Model (RTM) coupled to Michaelis–Menten / Monod growth.	cm–m	Daily
		Gene-centric model.		
		Bioclimatic model.		
Glacial meltwater and fjord productivity.	Hawkings *et al.* (2015), Meire *et al.* (2015)	Ocean/fjord biogeochemical model.	km^3^	Daily
Permafrost, wetlands, soils and tundra (ecosystem processes, methanogenesis and methane oxidation).	Panikov (1999), Bradley, Singarayer and Anesio (2014), Chong, Pearce and Convey (2015)	Depth-resolved Reactive Transport Model (RTM) coupled to Michaelis–Menten / Monod growth. Gene-centric model.	Plot (cm)—catchment (10^3^–10^6^ m^3^)	Daily–decadal
		Bioclimatic model.		
		Fitted model, SEM.		

The models presented in Table 3a are transferable, and could in theory be developed further to simulate a range of Polar and Alpine ecosystems, for example snow, cryoconite holes and lakes (Table 3b). For example, the short-term dynamics of microbial growth in a snowpack can be constrained by the same mathematical expressions that describe bacterial growth in a soil (Monod kinetics, *Q_10_* formulation, light limitation etc.). Statistically-based models, such as SEMs, rely heavily on the quality of the data from which they are constructed, and there is a need for complete, robust datasets encompassing multiple observations to explain system dynamics. For example, Schnecker *et al.* (2014) were able to determine, through fitted models, the controls on enzyme activity in Arctic soils and explain low decomposition rates of stored carbon. As identified in Table 3b, assuming comprehensive and high-quality datasets, statistical models could be applied to a range of questions in Polar and Alpine microbiology, including the spatial and seasonal dynamics of microbial communities in soils and tundra (Schadt *et al.*^2003^; Lipson and Schmidt 2004; Lazzaro, Brankatschk and Zeyer 2012; Chong, Pearce and Convey 2015).

Process-based models have also been applied, in context with Polar and Alpine microbiology, on ecosystem scales including ocean and ice-sheet basins, as well as over longer temporal scales (tens of years to many thousands of years) (Manizza *et al.*^2009^; Wadham *et al.*^2012^; Wieder, Bonan and Allison 2013; Schnecker *et al.*^2014^) and across latitudinal gradients, improving the accuracy of predictions in high-latitude regions (Arnosti *et al.*^2011^). These models are generally developed to study biogeochemical transformations and fluxes of carbon and macronutrients on the system-scale and to test the significance of certain processes for global biogeochemical cycles and climate. For example, Wadham *et al.* (2012) further developed and applied a well-established 1D numerical hydrate model (Davie and Buffett 2001) to explore the plausibility and potential size of a methane hydrate reservoir derived from the biogenic production of methane under the Antarctic ice sheet. Model development and scenarios were informed by experimental observations of the methane produced from microbial activity in sub-glacial sediments collected from various glaciers. Using this combined model-data approach, they demonstrated that there is potential for methane hydrate accumulation in Antarctic sedimentary basins, and the magnitude of methane stocks depend on the rate of microbial organic carbon degradation and the conditions at the ice–bed interface. This study extrapolated a series of local scenario applications to the entire Antarctic ice sheet, and over a timescale of thousands to millions of years. These larger scale biogeochemical models or upscaling strategies can be applied to a dynamic range of Polar systems where microbial processes are known to play an important role, including Arctic tundra ecosystems, and supra- and sub-glacial ecosystems including lakes underneath the Antarctic ice sheet (Table 3b). However, because most of these system-scale models emerged from the field of (bio)geochemistry, they often include an implicit rather than an explicit description of microbial biomass. Global scale modelling of microbial dynamics has been shown to improve the predictions of the Community Land Model (CLM) soil model (by including Michaelis–Menten kinetics in soil carbon pools), where latitudinal gradients are implicitly accounted for by temperature and enzyme kinetics (Wieder, Bonan and Allison 2013). An explicit description of microbial biomass in large-scale model applications is, in theory, possible, but model predictions would remain partly theoretical because laboratory data and field observations required for parameterization, calibration and testing are generally scarce. As a consequence, there is a clear need for microbiologists to inform future model development.

It has been shown that including accurate representations of non-linear metabolic processes such as Michaelis–Menten and temperature dynamics can improve the predictions of biogeochemical models (Wieder, Bonan and Allison 2013) and drastically affect simulated environmental outcomes (Bush *et al.*^2015^). The complexity of microbial dynamics in the next generation of Polar and Alpine microbial models will ultimately fall somewhere between first-order descriptions (whereby non-linear dynamics may be ignored for complex and unconstrainable processes) and complex non-linear mathematical descriptions (e.g. Wieder *et al.*^2015^) (which may improve predictions but require detailed data and prior understanding). Thus, in order to know what level of complexity or simplification is appropriate for a specific question, the modeller must consider that the assumptions being made (e.g. simplified linear processes) are not likely to lead to inaccurate predictions, and that complex mathematical formulae are fully integrated with and supported by independent empirical observations and understanding (e.g. Sierra, Malghani and Muller 2015).

## COMMON CRITICISMS TO MODELLING

All models are an imperfect representation of a complex reality. They should be viewed as a work in progress, and should be constantly re-evaluated and tested in the context of the evolving mechanistic understanding of these environments. The commonly quoted aphorism ‘all models are wrong, but some are useful’ (Box 1979) articulates that although an imperfect (or wrong) representation of reality, a suitable model applied to a specific question can be extremely useful. In fact, a more complete quote of Box's viewpoint: ‘Remember that all models are wrong; the practical question is how wrong do they have to be to not be useful?’ is probably a better encapsulation of this idea. Nevertheless, the use of models is still criticized and questioned (Pilkey and Pilkey-Jarvis 2007; Abraham 2009). Here, we aim to address some of the most common criticisms of mathematical models and present counter arguments that recognize both the limitations and power of model approaches specifically applied to Polar and Alpine systems. We do this in the hope that we can enthuse microbiologists working in Polar and Alpine regions to consider, develop and use integrated model-data approaches to explore the microbial dynamics of cold ecosystems.

### Models are too simplistic

The most important intellectual challenge in model development and application is the process of simplification. From a microbiologist's perspective, it is often difficult to accept that neglecting rather than incorporating every detail of a certain process results in a more useful model. The most complex model is not by default the most useful to answer a specific question. On the other hand, according to Einstein and following Occam's razor, a model should be as simple as possible, but not simpler. All biological processes in an ecosystem can be described mathematically, given some degree of simplification. However, not every known process will be important to answer the specific research question asked (there will also inevitably be processes occurring that we do not know about and thus cannot include). Furthermore, there is little to be gained from a complex description of a process if parameters cannot be constrained on the basis of available data. For example, complex physiological traits such as dormancy are meaningfully expressed in simple mathematical models of Arctic and Alpine soils by a single fixed parameter (Bradley *et al.*^2015^) or by Monod-type kinetics (Blagodatsky and Richter 1998). Ultimately, models should be designed to answer the specific questions as accurately and with as much confidence as possible. Thus, the simplifications that are inherent in model development can be thought of as one of the greatest strengths of modelling.

### Models cannot represent the diversity of a microbial community

It would be both impossible and unnecessary to incorporate the true magnitude of natural microbial diversity into a mathematical model. Instead, the level of detail that is adequate to represent microbial diversity (e.g. taxonomic rank) must vary depending on the research question. Whilst taxonomic based classification (e.g. species) is considered the most natural unit to describe the diversity of microbial communities, modellers may choose to organize, distinguish and classify microbial communities based on functional traits. Both approaches have been used in Polar models for different purposes. For example, the Stapleton *et al.* (2006) soil model distinguishes microbial communities according to phylum in order to represent predator–prey interactions between trophic levels. On the other hand, many processes associated with nutrient transformations involve microbial interactions where the grouping of certain species into functional groups can be useful for modelling purpose (e.g. sulphur oxidizers and nitrogen fixers) (see e.g. Bradley *et al.*^2015^). Additionally, population heterogeneity can be rigorously investigated using individual-based modelling approaches (e.g. Resat *et al.* 2012), which may be useful in the context of Polar environments, for example, to ascertain how unique microbial communities self-arrange and structure themselves over spatial gradients observed in Antarctic soils (Chong, Pearce and Convey 2015). Empirical characterization based on field sampling, after all, only provides a snapshot of the heterogeneity of a microbial community. Models, on the other hand, can be used to make predictions of microbial taxonomic and functional structure that extend far beyond the current range of possible observations.

### Biological systems are too chaotic to be constrained by models

Biological systems are, by their nature, inherently variable in time and space. This leads to heterogeneities that span scales, making datasets challenging to interpret, understand and draw inference from, and it is often difficult to interpret the signal through the noise. Models can be designed to be deterministic, but stochasticity can also be introduced to their formulations (e.g. Corradini, Normand and Peleg 2010; Dini-Andreote *et al.* 2015). Deterministic modelling (e.g. Baranyi and Pin 2001) is better suited to studying clear regular processes and causation, such as the structure of benthic microbial mats in ice-covered lakes (Zhang *et al.*^2015^). Alternatively, stochastic modelling (e.g. Baranyi 2002; McKellar 2002) may be more appropriate for population studies whereby the fate of individuals is not strictly determined but needs to be described by probability, frequency and variance, such as the stochastic nature of soil moisture in the Antarctic Dry Valleys, and the resulting heterogeneous microbial community composition (Zeglin *et al.*^2011^; Niederberger *et al.*^2015^).

### Models cannot deal with scale

Scale is an inherent problem in all aspects of environmental microbiology. The same generalisations in empirical studies have to be made in model building, and processes that dominate at the microscopic scale must be re-parameterized so that they are applicable on a coarser spatial scale. Simulating how microbial communities vary at different spatial scales is important in correlating diversity with environmental characteristics, in order to understand diversity hotspots (such as in Antarctic fjords (Grange and Smith 2013) and to test hypotheses about dispersal and colonisation (such as the airborne dispersal of soil organisms in the Antarctic Dry Valleys (Bottos *et al.* 2014b; Gonzalez *et al.*^2012^). The upscaling of small-scale processes is not likely to respond in a linear fashion (Schimel and Potter 1995) and thus may lead to uncertainties, and this must be kept in mind when interpreting model output. Simplifications and upscaling in mathematical models should be informed by bottom-up knowledge (see e.g. Murphy and Ginn 2000). A critical point is to ensure that the detailed, very specific knowledge from decades of microbiological research feeds into model development. However, that requires the willingness of microbiologists to take necessary steps in simplification and to communicate this knowledge to a modeler.

### Models are too heavily parameterized

Model parameters can be constrained either on the basis of theoretical considerations or through site-specific field and laboratory observations. However, this does not necessarily imply that all model parameters have to be constrained directly by theoretical considerations or observations. Many microbial or microbially-mediated processes such as microbial growth and organic matter decay are controlled by a complex interplay of different factors such as, for instance, light, temperature, thermodynamics, moisture availability and community structure. Because the significance of these different factors in controlling certain processes is still a matter of debate, models often do not explicitly account for all of these factors individually. In this case, model parameters derived from fitting observations or from environment-specific laboratory experiments implicitly account for the neglected factors (e.g. Blagodatsky and Richter 1998; Blagodatsky *et al.* 1998). Furthermore, model sensitivity analysis shows whether a given parameter has a strong effect on the dynamics and output of the model. In the case that its parameters are highly sensitive in the plausible range that has been established, experimental work can be designed to specifically constrain it (e.g. Blagodatsky *et al.* 1998). In the case that a parameter is poorly constrained but has a negligible effect on model output, this shows that the model can still be useful regardless of the uncertainty in this parameter.

### Models cannot be constrained by suitable observations

No matter what degree of complexity is built into a model, its usefulness or performance has to be tested, usually by comparing model output to observations. Year-round data in Polar and Alpine regions may not be of sufficient quality or scope to rigorously test a model, under the entire range of plausible environmental conditions. For instance, during the winter season, inaccessibility of field sites inhibits (or permits only periodical) sampling. Furthermore, low biological activity and biomass makes measurement and genomic characterization challenging. However, models that are validated with data from the summer (e.g. Bradley *et al.* 2015) can be run over the winter to predict the dynamics of the winter season for which observations are lacking. Such validated models are probably the best step towards understanding the dynamics of data-poor systems such as the winter in Polar and Alpine regions.

### Different models come to different answers

The model design process is subjective, requiring judgement and decisions that are generally guided by personal knowledge, background and experience. Consequently, the model building process is not unique. There are many fundamental differences between numerical models (see Table 2). Moreover, even within one certain approach to modelling (e.g. process-based modelling), processes might be formulated differently, potentially affecting model output. However, these different models and their results can be used to test not only our understanding and ability to represent the system numerically, but also test different mechanisms, causations and correlations in the natural system. Comparing differences in model output can provide valuable insights into the significance of single processes or the appropriateness of a process formulation (see for instance climate model intercomparison projects e.g. Taylor, Stouffer and Meehl (2012)). In addition, largely different model approaches such as simulation versus empirical models (e.g. direct representation of processes and mechanisms versus fitted mathematical expressions) often emerge from different research questions. Wieder, Bonan and Allison (2013) and Schnecker *et al.* (2014) both use models to investigate microbial dynamics in soils on a regional to global scale; however, the models they use (process-based versus statistical) differ according to the nature of the research question (capturing fluxes, quantifying rates and predicting future dynamics versus exploring spatial patterns and controls). Thus, different models, rather than coming to different answers, provide different insights.

## FUTURE OUTLOOKS

It is widely recognized that microbiology is and always has been a technology-driven science, from the invention of the microscope to the development of next-generation gene sequencing. Mathematical modelling is an underexploited resource for microbiologists working in Polar and Alpine ecosystems. Field and experimental approaches yield data and findings that feed into model design, but similarly, model design provides new insight into field and laboratory experiments that will shed new light on poorly understood processes (Fig. 1). At present, for example, there is a clear divide between modelling efforts (e.g. Stapleton *et al.*^2006^; Wadham *et al.*^2012^) and genomic studies (e.g. Pearce *et al.*^2012^) in Polar and Alpine systems. Yet there is much to be gained, as shown by Reed *et al.* (2014), by integrating these fields and designing models that can incorporate genomic data.

Models present a unique opportunity to expand knowledge in Polar and Alpine microbiology by

- analytically testing hypotheses that arise from observations;
- extrapolating, interpolating and budgeting processes, rates and other features to explore beyond the possibility of empirical observation;
- disentangling process interplay by examining the dynamics of working model formulations, and false models that provide useful negative results;
- exploring sensitivity (e.g. to amplified climate change in Polar regions (Serreze *et al.*^2000^)), making predictions and guiding future work;
- generating knowledge and serving as a platform of interdisciplinary knowledge synthesis; and
- quantitatively assessing the resilience of the Polar and Alpine microbiomes to natural or human-induced environmental changes.

We therefore advocate that future field and laboratory studies carefully consider how measurements are made such that data collected can be used directly in strengthening model design and validating predictions in the future. This includes an appreciation of whole-system budgets including inputs (e.g. allochthonous deposition in snowfall) and outputs (e.g. leaching via snowmelt), and data that can easily be put into context with model output (i.e. with appropriate units). Data should be reported in a homogeneous manor wherever possible, and where appropriate must indicate time, which is essential when rates are to be derived. Laboratory experiments are useful means to determine sensitive parameters and bridge the model-data divide, fostering collaborations on the design of conceptual models, thinking quantitatively, developing meaningful upscaling strategies and generic frameworks for parameterization of mathematical models of Polar and Alpine microbial communities.

We expect the role of numerical modelling in microbiology-focussed studies in Polar and Alpine regions to increase in the future, as technological capacity improves, data accumulates and understanding of the processes that govern these systems improves. However, as yet, the potential role of models is largely unrealized. Microbiology research is an inherently quantitative science, and will continue to become so. The simplifications and approximations inherent to numerical models that draw criticism should be seen as an opportunity to synthesize knowledge by critically discussing modelling concepts, the meaning of terms, criteria of relevance, identify knowledge gaps and ultimately provide new insights to complex biological processes in Polar and Alpine systems in future research.
